# “Alas … my sickness becomes my family's burden”: A nested qualitative study on the experience of advanced breast cancer patients across the disease trajectory in Indonesia

**DOI:** 10.1016/j.breast.2022.04.001

**Published:** 2022-04-04

**Authors:** Yayi Suryo Prabandari, Wika Hartanti, Syafriani, Mentari Widiastuti, Riani Witaningrum, Susanna Hilda Hutajulu, Matthew John Allsop

**Affiliations:** a Department of Health Behavior, Environment and Social Medicine, Faculty of Medicine, Public Health and Nursing, Universitas Gadjah Mada, Indonesia; b Center of Bioethics and Medical Humanities, Faculty of Medicine, Public Health and Nursing, Universitas Gadjah Mada, Indonesia; c Center of Health Behavior and Promotion, Faculty of Medicine, Public Health and Nursing, Universitas Gadjah Mada, Indonesia; d Division of Hematology and Medical Oncology, Department of Internal Medicine, Dr. Sardjito General Hospital, Yogyakarta, Indonesia; e Division of Hematology and Medical Oncology, Department of Internal Medicine, Faculty of Medicine, Public Health and Nursing, Universitas Gadjah Mada/Dr. Sardjito General Hospital, Yogyakarta, Indonesia; f Academic Unit of Palliative Care, Leeds Institute of Health Sciences, University of Leeds, UK

**Keywords:** Breast cancer, Qualitative, Indonesia, Chemotherapy, Illness trajectory

## Abstract

**Introduction:**

Limited research exists exploring the experience of living with advanced breast cancer in Indonesia. We sought to explore the narratives of women with breast cancer across the illness trajectory to understand their experiences from diagnosis to accessing and undergoing cancer treatments to inform the development of cancer care.

**Methods:**

A nested, exploratory study adopting a qualitative approach. We conducted in-depth face-to-face interviews with women living with advanced breast cancer in Yogyakarta, Indonesia. We purposively sampled participants by age, education and marital status. All interviews were transcribed verbatim with thematic analysis used to identify, analyse and report patterns and themes within the data.

**Findings:**

Four main themes were derived: 1) Early experiences, prior to accessing health care; 2) Navigating the system to access treatment; 3) Enduring chemotherapy and advancing disease, with crucial family support; 4) Seeking normalcy and belief in treatment. From initial symptoms through to undergoing treatments, the experience of participants was punctuated by barriers and challenges.

**Discussion:**

Presentation delays were driven by dismissing initial symptoms, seeking alternative medicines, and fear of surgery. Access to healthcare required participants to contend with long-distance travel to facilities, tiered and convoluted referral processes, and adverse effects and financial impact of treatments. Individual determination, belief in God, and the role of families were critical throughout the disease trajectory. Adopting a focus across the disease trajectory facilitated the identification of enduring and persistent challenges to care delivery that can inform targeted development and optimisation of care delivery for women with breast cancer.

## Highlights

• This is the first exploration of breast cancer patients' experiences across the disease trajectory in Indonesia.
• Patients endure convoluted referral processes, long distance travel, and commonly experience financial burden and severe side effects.
• Family play a critical role in providing physical, psychological and financial support during treatment.
• Healing is understood as resuming normalcy in daily activity, mobility and independence rather than cure.
• Focusing across the disease trajectory facilitated the identification of enduring and persistent challenges to care delivery that can inform targeted development of care delivery.

## 1 Introduction

In 2020, there were an estimated 10 million cancer deaths globally, with 58.3% occurring in Asia [1]. Female breast cancer is the most commonly diagnosed cancer globally [1] with some of the highest breast cancer incidence and mortality rates occurring in Asian countries including Indonesia [2,3]. In the context of Indonesia, the age-standardized incidence and mortality rates of breast cancer are 44 and 15.3 per 100,000 population, respectively [4]. In the country, breast cancer is mostly diagnosed at later stages [5,6] with low survival rates [7]. From limited research, patient and service-level factors are being identified that may influence the timing of interaction with health services and the common late presentation among breast cancer patients in Indonesia. For patients, delays in help-seeking are associated with the use of traditional or alternative treatments to alleviate breast cancer symptoms [8,9]. The economic status of patients is also associated with the timing of presentation to healthcare [10,11]. For example, whilst cancer management is available and can be supported through the national universal insurance program, additional out-of-pocket expenses can be incurred (such as transport, accommodation, and logistics) which can inhibit patient presentation to health facilities [10,11]. At the health service level, delays can be influenced by both health professionals and the intricate, tiered referral pathways from diagnosis to treatment [11].

The concept of an illness trajectory has been explored previously for people living with chronic illnesses, such as advanced cancer patients [12]. A trajectory can provide a framework for exploring different phases of a disease as they are experienced by people, from the initial identification of symptoms through to the advanced stages and dying. From existing research, there is emerging evidence to suggest that Indonesian patients with breast cancer encounter difficulties throughout the trajectory of their disease, from initial symptom identification to help-seeking and undergoing treatment. When breast cancer patients initially experience symptoms, they often first seek opinions from relatives and peers, and mostly present at health care facilities when their conditions have worsened [11]. Following diagnosis, breast cancer treatment processes have also been reported to be complex and intertwined within the sociocultural context [10]. However, Indonesian women's experience of accessing and receiving care for breast cancer remains understudied, with research to date focusing on discrete fragments of their illness. Studies across countries signify the need for a holistic portrayal of breast cancer patients' journey over the course of diagnosis and treatment to understand the broader implications of being diagnosed with breast cancer [6]. This research aims to address this gap in the literature for Indonesia, exploring the narratives of patients with breast cancer to understand their experiences from diagnosis to accessing and undergoing cancer treatments.

## 2 Material and methods

### 2.1 Setting

This exploratory study adopted a qualitative approach with women living with advanced breast cancer in Yogyakarta, Indonesia. This nested study was undertaken as part of a larger cohort study on chemotherapy services at Dr. Sardjito Hospital, Yogyakarta, Indonesia. The parent study was “*Analyses of chemotherapy toxicities in breast cancer patients, predicting risk factors and the influence on patients’ survival and quality of life”*. All participants in this study received first line chemotherapy.

### 2.2 Sampling

Purposive sampling was adopted to recruit patient participants from the parent study. Participants were eligible if they had metastatic breast cancer, were not deemed too ill by the medical team to participate and had been recruited to the parent study within 12 months prior to data collection. A purposive sampling frame identified potential participants with variation according to age, education level and marital status. We sought to recruit up to 20 participants to provide a diverse range of perspectives and experiences across the breast cancer disease trajectory from our target population. The intended sample size was informed by the concept of data saturation given the purposive sampling approach adopted in this study [13]. Data saturation involved monitoring incoming data from interviews to determine a point at which little or no new information relevant to the study objectives was emerging. This was achieved through the interviewer summarising key aspects raised by a participant following an interview, which was subsequently discussed with the wider research team. Key points were described and compiled to support monitoring of interview data during the data collection period. Although the extent of new information being identified reduced after the first ten interviews, we continued to the target sample to ensure maximum diversity across characteristics of the sampling frame.

### 2.3 Data collection

Data collection started from June to November 2019 using semi-structured face-to-face interviews. Project-specific consent procedures were developed for this study, including detailed information sheets relating to the planned interviews and an additional consent form. Participants were approached through screening of parent study participants. Those meeting the eligibility criteria were approached by a member of the research team to introduce this study. Potential participants were provided with an information sheet detailing involvement in a nested qualitative component of the parent study. Those who agreed to participate completed a consent form prior to participation. All participants were interviewed once and alone in a private room in the cancer clinic at Dr. Sardjito Hospital, Yogyakarta. Topic guides developed by the research team were used to guide exploration of participants’ experiences of first symptoms, initial contact with health care providers and other sources of support, factors influencing the decision to seek support, and experiences of undergoing treatments. All interviews were audio-recorded. Following an interview, researchers made notes which were discussed as part of research team meetings to inform the analysis process and monitor data saturation. All interviews were later transcribed for analysis supported by OpenCode software version 4.03.

### 2.4 Data analysis

**Fig. 1:**
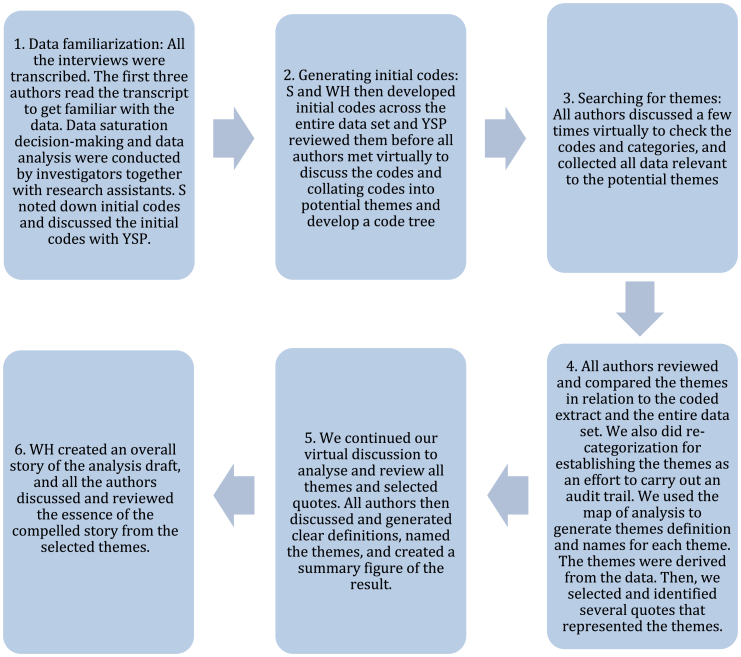
Overview of steps taken during thematic analysis.

We adopted thematic analysis to identify, analyse and report patterns and themes within the data. We outline the process adopted during analysis including the development of themes in Fig. 1. We followed the six stages of thematic analysis as outlined by Braun and Clarke [14]. This included generating initial codes from participant transcripts, grouping codes with similar semantic meaning, from which codes were grouped together to form themes. Themes were formed using explicit or surface meaning of transcript content rather than through interpretation by the research team. Themes were discussed and refined by the research team until consensus was reached. We then adopted a thematic network approach [15] to develop a schematic depicting how principal themes and patterns aligned with the original questions. The schematic was developed iteratively through discussion by the research team. The study is reported in accordance with the Consolidated criteria for reporting qualitative research (COREQ) checklist [16].

## 3 Results

In total, 20 participants with metastatic breast cancer participated with a description of participant characteristics presented in Table 1. Interviews lasted a median of 31.41 min.

**Table 1:** Characteristics of participants.

Participant informants (total n = 20)		n	%
Age	40–49	10	50
	50–59	6	30
	60–69	4	20
Highest level of education	Primary/junior high school	14	70
	Senior high school/university	6	30
Marital status	Single	1	5
	Widowed	1	5
	Married	18	90
Number of children	0	1	5
	1	3	15
	2	12	60
	3 or more	4	20
Status of employment	Housewife	10	50
	Employed/self-employed outside the home	9	45
	No job	1	5
Time since diagnosis (per 2019)	0 year	6	30
	1 year	12	60
	2 years	0	0
	3 years	2	10
Metastasis status	Oligo metastasis	15	75
	Multiple metastases	5	25
Stage at initial diagnosis	Stage III (locally advanced)	3	15
	Stage IV (metastatic disease)	17	85

**Fig. 2:**
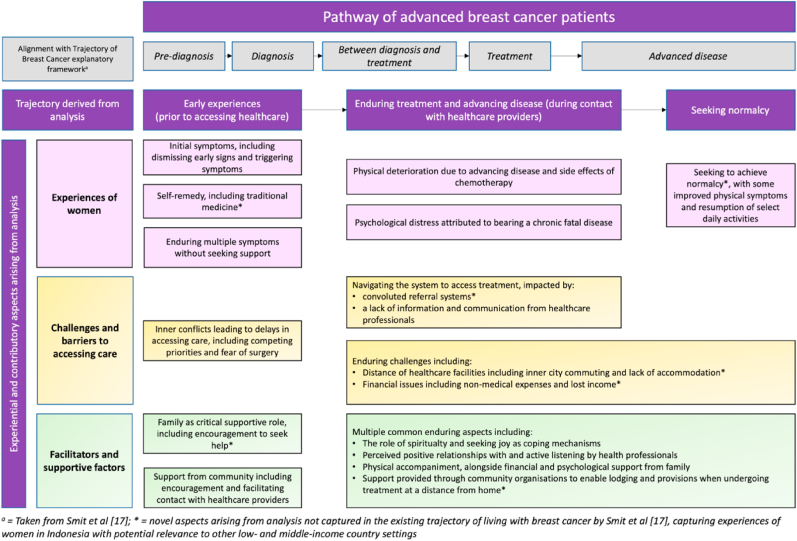
Thematic network schematic to represent key findings from the analysis.

Four main themes were derived through analysis: 1) Early experiences, prior to accessing health care; 2) Navigating the system to access treatment; 3) Enduring chemotherapy and advancing disease, with crucial family support; 4) Seeking normalcy and belief in treatment. We have included quotations to illustrate findings across the derived themes in Table 2. A schematic depicting how principal themes and patterns aligned with the original questions and with each other is shown in Fig. 2.

**Table 2:** Illustrative quotes for the themes and sub-themes.

Sub Themes		Illustrative quote
**Theme 1: Early experiences, prior to accessing health care**		
**Early signs and self-care adopted during initial breast cancer symptoms**	1	“When I found the lump, I did not do anything. I thought it was not that serious. I did not feel pain. Even when I perform daily activities, I do not feel pain or fatigue, no.” (Participant 13, 56 years old)
	2	“(initially) I wasn't too concerned about the disease, as I've consulted with the herbalist, and asked if the disease could be managed in the herbal clinic and whether we needed to get treated at the hospital. (The herbalist said) ‘with God's will, we can treat the disease here, no need to go to the hospital.’ So, I (felt assured and) had no concern. (Participant 10, 46 years old)”
	3	“I ignored it for about 2 years. Then the lump got bigger and bigger to the point that I started feeling pain. The pain spread to this and this part. I was terrified, (only) then I went to Hospital B.” (Participant 15, 54 years old)
	4	“… I don't feel it, ma'am … then … here … my heart is like being squeezed, you know … it feels congested … I think it's because of my stomach acid … The first time I knew that … kept going to the district hospital once, twice, three times … but still didn't get better. Then I asked for a referral to a provisional general hospital. Then you know … I was on the X-ray … it turned out that my lungs were full of fluid … the doctor said the fluid was coming out of my breast … which … had hit the motorcycle handlebar in the past”. (Participant 18, 47 years old)
	5	“I took alternative medicine … there was no improvement, Ma'am. The practitioner guaranteed this, this, this, but there was no improvement. One of them sold the medicine for one million [rupiah] per package … but it's pricey.” (Participant 08, 56 years old)
**Inner conflicts around fear of surgery and competing priorities**	6	“I was asked to do surgery, but I said, I don't want to, I was afraid. I just ignored it and kept taking herbal medicine for 3 years” (Participant 03, 64 years old)
	7	“(At that time) my children were still very young and at school age. I thought children's school had to be prioritized more, and maybe I could deal with my conditions later (after they finished school)”. (Participant 10, 46 years old)
**Family and wider social supporting roles in encouraging and facilitating contact with healthcare services**	8	“(I thought) it was only a lump, not a big deal. But after two months my blood pressure was low, so my daughter told me to just go to the hospital, get medical consultation and examination, and if the doctor said to get breast surgery, she told me I had to comply. I agree and follow her (lead), as what's important is that I can be healed”. (Participant 12, 56 years old)
	9	“My children always encourage me to undergo treatment, although I said I felt better, I should not go, I make my children so busy to take care of me …. for example, I felt pain on my shoulder, then my children ask me to go to the hospital because they worry that the pain was related to my breast” (Participant 07, 58 years old)
	10	“In the past, my face was very pale. When I met my neighbour, she was worried about my health. At that time, I said, I was just a little dizzy, had a headache and had a fever. After that my neighbour visited me and persuaded me to go to the Puskesmas [Primary Health Care] for treatment. I was not mentally ready for treatment, I was afraid. Next time, the Puskesmas staff visited me and explained various things. Thus, I agreed to go to the Puskesmas“. (Participant 10, 43 years old)
**Theme 2: Navigating the system to access treatment**		
**Convoluted health care referral systems**	11	“… When I felt pain, I told my husband to take me to Puskesmas that night. Then he took me to Puskesmas and I got referred to a district-level hospital. Then, I got referred to a provincial-level hospital.” (Participant 02, 53 years old)
	12	“The physician at Puskesmas said, ‘Ma'am, you have to … you need to go to the hospital. I will make a referral. Where do you want to be referred to?’ The doctor asked me to choose between several hospitals in Jogja. I chose one hospital (Hospital P). But the doctor (internist) at Hospital P said the lump needed to be removed with surgery. It cannot be treated with medication alone. Medication will not be suitable. Just undergo surgery as soon as possible (the internist referred the participant to a surgeon)” (Participant 03, 64 years old)
**Impact of travel due to distance of health facilities**	13	“I had experienced bad physical conditions during my trips to the hospital. My haemoglobin level was only 5,8. During the trip, I vomited blood … I came from Klaten [a neighbouring city about 45 min from Yogyakarta] which is quite far. (Participant 06, 41 years old)
	14	“The transport [travel to the hospital] was far. It costs three hundred thousand rupiahs [equivalent to 30 US dollars] for one trip, and six hundred thousand rupiahs [equivalent to 60 US dollars] for round-trips … for every treatment visit, I had to stay here for 2–3 days, and initially, I spent two hundred thousand rupiahs [approximately 20 US dollars] per night [for the accommodation]. Luckily I had a nephew here who now provided me with room to stay for free with every hospital visit.” (Participant 07, 58 years old)
**Caregiver lost income due to extent of support required**	15	“My husband was so supportive of my treatment that he had to leave his job. He used to work at the airport, but because of taking care of me, he quit his job and is now working non-permanent jobs … as he'd need to accompany my visit to the hospital 2–3 times a week, for chemotherapy, routine check-ups, and picking up the medication.” (Participants 10, 46 years old)
**Health professional communication**	16	“Before the current doctor, we had encountered a doctor who said harsh things to us. That doctor said this disease couldn't be cured anymore, it was already at stage 4, the terminal stage, incurable, and told us to just go home. Sometimes we encountered doctors who were unfriendly, this doctor even said bad discouraging words to a patient who was severely ill.” (Participant 01, 61 years old)
	17	“Previously, I was referred to XX Hospital. The service was not good. One doctor served several patients. I am an impatient person. Imagine, at that time, I had just finished surgery, then I had to travel to XX Hospital at 3 a.m. by car with my weak condition. When I got there and met the doctor, I was only given painkillers and got the wound bandage changed. If it was only for changing the bandage, I could do it myself at home. I said to the doctor that I was going to this hospital for chemotherapy. But the doctor scolded me and said “Enough! I don't need your argument”. At that time, fortunately, I got advice from another doctor to change the referral to this current hospital “. (Participant 18, 47 years old)
**Theme 3: Enduring chemotherapy and advancing disease, with crucial family support**		
**Enduring experience of side effects experienced when undergoing chemotherapy**	18	“After chemo, I felt nauseous, I didn't want to eat, then my nails turned black and my skin was scaly like fish skin.” (Participant 09, 63 years old)
	19	“After the first chemo, I felt major pain, that I couldn't walk normally like I used to prior to chemo, (after the first chemo) I had to walk very slowly and carefully. The doctor even prescribed me that orange morphine.” (Participant 05, 55 years old)
	20	“When I first went to Sardjito Hospital, I cried when I looked at the patients here. It was terrifying. Some patients' skin seems to have darkened, and I cried. ‘Oh, do I have to die so soon? I am not ready to leave my grandchildren.’ For a month I kept crying. Well, Ma'am, it's stressful … to be diagnosed with cancer. ‘Oh Allah, will I be able to go to the mosque during the fasting month next year?’ At the time, I cried so hard until everyone in the mosque came to me. They tried to comfort me, ‘Ma'am, you need to keep believing that you will recover.’ Will I be here during the fasting month next year? It's so scary. Oh God, please extend my lifespan. I am not ready to die. I am full of sin. And I still want to take care of my grandchildren.” (Participant 05, 55 years old)
	21	“I was desperate during the second chemo. I didn't think I could continue. I felt tortured, thought I'd rather die than suffer like that. (I didn't expect) the cure to my illness would feel torturous.” (Participant 03, 64 years old)
**Coping through spirituality and joy, alongside alternative therapy**	22	“Now, I no longer think about how long I live. I just want to do the things that make me happy. I sing, I listen to Wayang (traditional java performance) in order to sleep well”. (Participant 05, 55 years old)
	23	“I just want to be healed, that's it. Every night I read zikr [Islamic utterance/prayer] in order to ask Allah for recovery. Allah does not test humans beyond our ability, Mam. I believe it.” (Participant 06, 41 years old)
	24	“I got a massage after having surgery. I also used special oil from Surabaya. It felt hot when I used it. The oil is clear like eucalyptus oil. My husband's friend used the oil and her breast’ wound dried up quickly. I observed mine is getting smaller” (Participant 08, 56 years old)
**Family support, encouragement and company**	25	“I did the whole treatment process patiently. Sometimes, I feel sorry for my children who accompany me for treatment. I often say I want to quit the treatment so they won't be troubled. But my children encouraged me to continue treatment”. (Participant 09, 63 years old)
	26	“I am a housewife. My living expenses including meals are fully provided by my children. They cover medical expenses that are not covered by BPJS. Alhamdulillah, they are very caring, taking care of me wholeheartedly” (Participant 05, 55 years old)
	27	“I felt like complaining to God, and sort of asking God to end this (life) once and for all, because I pity my kids for going through troubles taking care of me. Alas. my sickness, becomes my family's burden. (In terms of) many aspects like financial, efforts, and time. But my children insisted that I needed to stay strong, have more patience. (Children said) this is just how it's supposed to be, like I used to take care of them as children, and now it's their turn to take care of me their mother. Those kids collectively motivated, supported and entertained me.” (Participant 03, 65 years old)
**Positive and motivational communication with health professionals**	28	“The doctor (here in this hospital) asked about my health condition, listened to my complaints, then the doctor explained the chemo schedule” (Participant 01, 61 years old).
	29	“Thank God, the service at this hospital is very good. I have been admitted and hospitalised here many times, so I know the nurses well. They are friendly and playful”. (Participant 04, 50 years old)
**Support from wider organisations**	30	“My nephew introduced me to Sedekah Rombongan (community-based charity foundation). It provided me with free lodging, and volunteers who took me to and from the hospital. It's already been a year and more that I've been assisted by SR. During the early treatment, it was very difficult, I couldn't commute, so I stayed at the SR lodging, free of charge. I didn't have the money to commute.” (Participant 04, 50 years old)
	31	“My religious (Quran recital) group of friends send encouragement and motivations. I am no longer able to join their group activities, because I'm focusing on my treatments, but they often visit me and pray for me.” (Participant 09, 63 years old)
**Theme 4: Seeking normalcy and belief in treatment**		
**Sense of improving physical symptoms** **And resuming daily activities**	32	“I am in good condition after chemo. Although I feel weak after chemo, my appetite is getting better. My friends said, my body is not as thin as before. I also feel that my face skin gets brighter and my nails are no longer black”. (Participant 03, 64 years old)
	33	“… I feel better after chemo. Before, I had to change wound bandages twice a day due to a lot of pus, now it's once a day and this pus fluid has reduced. Before I had a lot of breast pain, discomfort in various positions, and sleep disturbance. After the second chemo cycle, the pain diminished, and I could sleep better.” (Participant 05, 55 years old).
	34	“After several rays and chemo, I feel better and healthier and able to walk [at the beginning of the treatment I was still using a wheelchair]. Thank God (I feel) a lot of improvement. I was unable to walk, now I walk fine. I felt pain then, now no more pain. I feel a lot healthier, unlike the past times when many things hurt, but now I can even ride my motorcycle by myself. In the past, I couldn't even get up from bed nor eat without help.” (Participant 13, 56 years old)
**Confidence in self-management of condition**	35	“Even now, sometimes I take care of it myself, the hospital staff explains how to treat the wound myself. I bought wound treatment at the pharmacy. Morning and evening, I change the bandage and put on the underpad. Now the wound is getting smaller” (Participant 10, 46 years old).
**Willingness to persevere with treatment**	36	“I used to be very afraid to go to the hospital because I heard bad experiences from my neighbours and my friends. However, after I went through [chemotherapy] it turned out to be easy. After being referred here [Sardjito Hospital] my illness was immediately treated. There are many doctors here who are ready to help, medicines are also available. If I cannot see a certain doctor, there are other medical officers who may substitute. Now I am no longer afraid. I am happy, I want to be treated quickly because I want to get well soon. The important thing is to keep up the spirit and not think negatively. After knowing this disease, I went through all the doctor's advice. I'm not discouraged” (Participant 10, 46 years old).
**Preferences for health professional communication regarding their management**	37	“So, I just want to follow (doctor's suggestion) because I really want to be healed … (doctor said) it's important to get immediate treatment … (if I have to) get chemo then so be it … as long as I can get help (for my disease)” (Participant 18, 47 years old)
	38	“… I said this to the doctor ‘I am afraid of this disease [cancer]. So, doctor … if there is anything related to my illness, please just communicate it to my child … ‘. ‘Oh, alright’, said the doctor.” (Participant 16, 64 years old).
**Outcome determined by God Almighty**	39	“Well … I just surrendered … because Allah gave me the disease. So … I am ready for what will happen … the important thing is I pray. I am not afraid … nor sad … just surrender … to Allah. I will follow whatever the doctor decides to do … the important thing is that I am cured” (Participant 19, 47 years old).

### 3.1 Early experiences, prior to accessing health care

All participants initially dismissed their earliest symptoms, often experienced as a painless lump in the breast (quote 1). With the growth of a lump sometimes accompanied by changing breast shape, participants would typically explore self-care using herbal or traditional medicine approaches (quote 2). Most participants decided to seek medical care when symptoms worsened, including lumps becoming painful, changes in the shape or appearance of the breast, and increasing pain in other parts of their body, despite efforts to manage them (quote 3). Some participants endured multiple symptoms without accessing healthcare services, including difficulties with breathing and the feeling of a heavy chest (quote 4). Costs of medical care were perceived as unaffordable by participants, despite continuing to incur costs from traditional medicine providers with little or no improvement in their symptoms (quote 5). Alongside cost, additional factors influencing participants’ decisions regarding engagement with healthcare included personal, inner conflicts and the influence and encouragement of family. Participants were reluctant to access healthcare facilities due to fear and assumptions relating to surgery (quote 6), and for women with children, most felt their needs and concerns should be secondary to those of their children (quote 7). Facilitating factors that encouraged engagement included family members urging participants to do so for worsening symptoms (quote 8, quote 9) and neighbours (quote 10).

### 3.2 Navigating the system to access treatment

Participants reported difficulties with accessing support for their symptoms, often having to navigate lengthy, convoluted referral processes and enduring the necessary travel across long distances to larger facilities with the capacity to undertake diagnostic tests. Following numerous primary care consultations, a referral to a larger hospital facility occurred for diagnosis by medical specialists (quote 11), followed by further onward referrals to other medical specialists for treatment (quote 12). Multiple delays often took place between each point of access with health providers. Every trip affected and sometimes worsened participants' physical condition due to exhaustion and discomfort during transportation (quote 13). Participants who had no private vehicle would have to use public transportation. These commuting routines resulted in significant expenses for transportation and accommodation (quote 14). Some participants and family members even lost their income sources, as they had to take leave or quit their regular jobs to attend treatment sessions (quote 15). Following referral to specialists for treatment, participants often had to incur waits of up to one month for follow-up consultations and to commence treatment. Despite their efforts to access care, some participants reported feeling discouraged when met with criticism from doctors for presenting with breast cancer at a late, advanced stage (quote 16) and when seeking more effective management of their condition (quote 17).

### 3.3 Enduring chemotherapy and advancing disease, with crucial family support

Cancer-related problems reported by participants included physical deterioration due to late-stage cancer, side effects of chemotherapy, and psychological distress. All participants reported difficult experiences when undergoing chemotherapy with commonly experienced side effects including frequent nausea and vomiting, loss of appetite, and difficulties with eating, swallowing and retaining food despite feelings of hunger and thirst. Participants also reported losing weight and feeling fatigued whilst undergoing treatment (quote 18). Some participants experienced severe pain during chemotherapy which affected mobility and elevated blood pressure and required morphine for its management (quote 19). Participants also highlighted the high levels of distress caused when considering possible outcomes from treatment (quote 20), alongside being emotionally sensitive and dispirited, with a few feeling “like dying” when expressing their suffering (quote 21).

Supportive and protective factors were reported by participants which facilitated coping with treatment. At an individual level, participants coped with their illness through finding joy and spirituality, along with incorporating alternative treatments alongside the medical approach. Some participants sought to divert negative thoughts through leisure activities such as listening to music and activities that made them happy (quote 22). Participants also relied on their spirituality by praying and surrendering to God, holding the belief that cancer is a form of trial to be endured which strengthened hopes for recovery (quote 23). Participants also reported combining alternative treatments to speed up postoperative wound healing (quote 24).

Participants illustrated the critical roles of others. Participants' families often encouraged compliance and adherence to treatment courses. Family members maintained optimism to motivate participants to complete treatment programs, alongside physically accompanying them to hospital visits (quote 25). Family members would take care of participants' needs during and between treatment sessions, including keeping participants motivated and entertained throughout treatment. Participants explained that family roles extend into financial support, as families would collect funds to cover expenses, mostly non-medical, throughout the treatment program (quote 26). Whilst providing a crucial supporting role, participants were often conflicted with the level of strain this could place on family members (quote 27). Supporting that this conflicted with a sense of placing a burden on them. Supportive others included health professionals, who through friendly gestures, such as actively initiating communication and listening to participants' feelings and complaints, encouraged participants to continue treatment (quote 28, 29). Wider society-based initiatives and community-based charities were also reported to support participants through free lodging, amenities, and volunteers’ assistance during participant stays for treatment (quote 30) and forms of psychological support such as sending prayers (quote 31).

### 3.4 Seeking normalcy and belief in treatment

Participants reported that they and their families perceived a sense of healing that could be achieved through regaining a sense of normalcy in their daily lives. After completing chemotherapy, most participants in our study felt improvement in physical symptoms, such as a dried wound, regaining appetite and body weight, as well as experiencing improved sleep quality (quote 32, 33). Some participants also reported improved mobility including returning to walking unaided (quote 34). Participants also reported developing confidence and competence with self-management approaches, including treating their wounds at home without support from nurses (quote 35). Most participants reported becoming increasingly positive and motivated to persevere with medical treatments, as they felt more able to manage treatment courses that offered an opportunity for healing (quote 36). As treatment continued, clear communication of their disease and treatment by healthcare professionals facilitated retention of hope for some participants (quote 37). Other participants preferred doctors to communicate with their family on their behalf, particularly when too ill or in physical discomfort which could affect their comprehension of details being provided (quote 38). All participants expressed a determination to persist with and explore options for treatment as a route to improving their condition. Most participants believed that it was important to persevere with treatments and that the outcomes would be determined by God Almighty (quote 39).

## 4 Discussion

Our in-depth exploration of the experience of women with advanced breast cancer provides a novel depiction across the disease trajectory from initial symptoms, to presentation, and undergoing chemotherapy treatments. We highlight common delays in the presentation to health providers when initially experiencing symptoms that were later determined to be indicative of breast cancer. Waiting for symptoms to worsen and resorting to alternative medicines were often driven by psychological and economic factors. Once accessing health facilities for treatment of breast cancer, participants commonly had to contend with long-distance travel to facilities, tiered and convoluted referral processes, and multiple adverse effects and financial burden from undergoing treatment. Critical throughout these stages of the disease trajectory was the role of individual strength and determination, belief in God, and the role of families who were crucial advocates and sources of support. While living with advanced disease, access to treatment provided a sense of healing when participants felt they were regaining the ability to undertake aspects of daily activities as they sought to return a sense of normalcy to their lives.

Our findings provide the first report on the experiences of women with metastatic breast cancer that explores experiences across the disease trajectory in the context of a low- and middle-income country setting. Existing phases of a breast cancer pathway have been derived through a recent systematic review and meta-synthesis of qualitative evidence [17]. However, the pathway was derived from studies commonly focusing on a limited timepoint in the disease trajectory (e.g. receiving an early diagnosis of breast cancer, or having undergone breast cancer surgery) alongside highlighting the need for more research exploring the experiences of women from developing countries [17]. The concept of trajectory introduced by Strauss incorporates not just the ‘physiological unfolding of a patient's disease but the total organization of work done over the course of illness and the impact on those involved with that work and its organization’ [18]. The broader temporal focus presented in this study enables exploration and identification of both factors that are limited to specific phases, and those that are enduring and persistent. Reported experiences of women in this study align with the key phases of a breast cancer pathway whilst contributing novel elements; the integral role of family, and the persistent challenges experienced relating to convoluted and complex referral processes across services and settings.

At a regional level, our findings correspond with existing literature from the South East Asia region whilst providing novel contributions for the context of Indonesia. Poor recognition of breast cancer symptoms is a cause of delayed presentation in Malaysia [19]. Furthermore, a low perception of disease severity upon discovering initial symptoms and tended to see symptoms as normal pain or signs of psychological distress aligns with experiences of Iranian women with breast cancer [20]. The response could be indicative of low health literacy among participants (i.e. the ability to understand and use information to make decisions about their health); the most common barrier to cancer diagnosis in LMICs [21]. For those receiving a confirmed diagnosis of cancer, feelings of fear, shame, denial and financial concerns were common among participants, aligned with previous work in Indonesia [22]. Surrounding participants from initial symptoms through to undergoing treatments were family. Family caregivers and members influenced decision making to engage with and seek support from health services. This included prioritisation of the need of their children over their own health, alongside the role of families in persuading participants to seek care. This aligns with Indonesian society; a country with strong social cohesion and regarded as one of the least individualistic societies globally [23,24].

Cancer control efforts span risk factor modification and prevention, early diagnosis, treatment, and palliation [21]. There is limited development across all stages in Indonesia. For prevention, programs are often implemented under *Puskesmas* (Primary Health Care), including an existing breast cancer awareness program initiated by the Indonesian Health Ministry; the *BCearly* detection program. *BCearly* targets primary prevention of breast and cervical cancers [25]. Government-led initiatives often occur alongside those led by non-governmental organisations (e.g. the Indonesian Cancer Foundation and oncology societies), charities, cancer support groups, and survivor societies. Campaigns generally seek to deliver public education by cancer experts and survivors, often targeting high-risk individuals (e.g. family members of cancer patients). Participants’ dismissing of symptoms and fears around accessing care evidence a need for further development and evaluation of current campaigns. Primary care is a key requirement of universal health coverage [26] but there is known inequity in service provision across Indonesia [27]. With the development and refinement of campaigns, there needs to be structural investment in primary healthcare to accommodate increased demand from women with breast cancer, irrespective of location. Following presentation to cancer care services, the experience of commencing and undergoing treatments was complex for participants. Unmet support needs were evident across three domains aligned with breast cancer treatment [28]; i) health system and information, ii) physical, and iii) psychological. All were prominent in this study, with adverse physical and psychological effects aligned with existing reports of trauma caused by intense pain, fear of death, and stress when undergoing breast cancer treatments in Indonesia [29]. There is, however, a lack of holistic care for people with advanced breast cancer in Indonesia, despite the need [30]. Critical areas of clinical importance that include physical symptom control, psychosocial support, physical activity, nutrition support, and advance care planning, could be supported through provision of palliative care. Whilst the Ministry of Health developed a palliative care policy in 2007, progress with implementation has been slow with very limited, disparate provision across the country.

We outline findings that can inform multiple routes to improving care for women with breast cancer in Indonesia. Firstly, health professionals reflecting empathy and good communication can improve the experience of care for patients; sincere motivational words can be perceived positively by patients with cancer in Indonesia [29]. Secondly, families need to be considered as key stakeholders in the development of future approaches that seek to improve the delivery of care for women with breast cancer. Family caregivers and members greatly influence decision making to engage with and seek support from health services, including participants reporting prioritisation of the need of their children over their own health, with family members continuing to play a key supportive role throughout treatment. Thirdly, once undergoing treatments participants reported a determination to endure and complete treatments. Exploring ways of providing support during treatment such as leveraging digital health approaches to support self-management [31,32] or adaptation of remote monitoring of adverse events [33] may provide valuable tools to augment the delivery and quality of care. Lastly, palliative care development to better meet the needs of women undergoing treatments and living with advanced disease should be prioritized through, for example, building capacity and increasing the palliative care workforce, creating care models that provide services in the community, and exploring integration across oncology care and other disease groups. There is also scope to use our findings to guide provision of both country- or region-specific care pathways, drawing on crossover in experiences of women with breast cancer in this study and those in other countries in Southeast Asia, such as Singapore, where similar factors (e.g. fear and a lack of information) have been identified as affecting initial presentation [34]. Devising tailored care pathways could guide Indonesia and countries in the region towards universal health coverage [35]; where achieved it is independently associated with decreasing breast cancer mortality [36]. Beyond Southeast Asia, this study highlights the value of exploring experiences across the disease trajectory to augment the evidence base for breast cancer care in low- and middle-income countries, providing an approach to understand both phase-specific and enduring factors to inform prioritisation for service development.

The study was conducted by a team of experienced researchers in the field of oncology, psychology, medical science, public health, and bioethics. We recruited participants that reflected diversity in age, education, marital status, occupation, time since diagnosis and stage at diagnosis. However, the study took place in one cancer centre in Indonesia and undergoing chemotherapy hence the results may not resemble the experiences of patients in other centres in the country and women experiencing other treatment modalities. Furthermore, ethical approval obtained for the study required all interviews to take place in the hospital setting and not in the home of participants. Subsequently, the research team was not able to follow up with participants in the community to seek further input from participants on the study findings to verify their accuracy in the portrayal of their experiences.

## 5 Conclusions

Delays in presentation to health providers when initially experiencing symptoms indicative of breast cancer were common across participants. From initial symptoms through to undergoing treatments, the experience of participants was continually punctuated by barriers and challenges. However, family members were a constant source of support and influence across the illness trajectory. Family caregivers should be considered key stakeholders in the development of approaches to improve the delivery of breast cancer care across the stages of risk factor modification and prevention, early diagnosis, treatment, or palliation. While interventions are being developed across each of these four elements of cancer control in Indonesia, there is a dearth of evidence to indicate their acceptability and effectiveness. Future research spanning the disease trajectory could facilitate the identification of enduring and persistent challenges to care delivery that can be targeted to guide the development and optimisation of support for women with breast cancer.

## Author contributions

YSP, SSH and MJA conceived the idea. Data collection was performed by SS, WH, YSP. Data analysis and interpretation, as well as manuscript writing and finalisation were performed by all authors.

## Funding

This work was supported by the Faculty of Medicine, Public Health and Nursing, Universitas Gadjah Mada (2019) multidisciplinary research scheme grant, received by YSP. SHH received funding from 10.13039/501100009509Kementrian Riset, Teknologi dan Pendidikan Tinggi Republik Indonesia (ID) (2018) to undertake the project. MJA received funding from Research England Quality-related Research GCRF from the University of Leeds (2019).

## Ethical approval

This study has been approved by the Medicine and Health Research Ethics Committee of the Faculty of Medicine, Public Health and Nursing, Universitas Gadjah Mada and Dr. Sardjito Hospital, Yogyakarta, Indonesia (reference number KE/FK/0417/EC/2018). A study permit was also granted by the Dr. Sardjito Hospital director.

## Declaration of competing interest

The authors declare no conflict of interest.
